# Does drug-induced sleep endoscopy change the surgical decision in surgically naïve non-syndromic children with snoring/sleep disordered breathing from the standard adenotonsillectomy? A retrospective cohort study

**DOI:** 10.1186/s40463-017-0190-6

**Published:** 2017-02-13

**Authors:** Malak Jamal Gazzaz, André Isaac, Scott Anderson, Noura Alsufyani, Yaser Alrajhi, Hamdy El-Hakim

**Affiliations:** 1 Division of Otolaryngology-Head and Neck Surgery, Department of Surgery, University of Alberta, Edmonton, AB Canada; 2 Faculty of Medicine and Dentistry, University of Alberta, Edmonton, AB Canada; 3 Department of Dentistry, Faculty of Medicine and Dentistry, University of Alberta, Edmonton, AB Canada; 4 Division of Pediatric Surgery, Department of Surgery, Stollery Children’s Hospital, Edmonton, AB Canada; 5 Division of Otolaryngology - Head and Neck Surgery, Umm Al-Qura University, Makkah, Saudi Arabia; 6 Department of Oral Medicine and Diagnostic Sciences, College of Dentistry, King Saud University, Riyadh, Saudi Arabia

**Keywords:** Drug induced sleep endoscopy, Sleep disordered breathing, Adenotonsillar hypertrophy, Decision-making, Adenotonsillectomy, Children

## Abstract

**Background:**

Adenotonsillectomy is the most commonly performed operation for pediatric snoring/sleep disordered breathing (S/SDB). However, 20–40% of patients will fail to improve. Drug-induced sleep endoscopy (DISE) may provide a more individualized surgical plan and limit unsuccessful surgeries. The aim of this study was to assess the impact of DISE on surgical decision-making in surgically naïve children with S/SDB.

**Methods:**

A retrospective observational cohort study was undertaken at the Stollery Children’s Hospital. Patients 3–17 years of age who underwent DISE-directed surgery for S/SDB between January 2009 and December 2015 were eligible. We excluded other indications for tonsillectomy and syndromic children. The primary outcome was the level of agreement between a DISE-based surgical decision and the reference standard based on the American Academy of Pediatrics (AAP) guidelines via un-weighted Cohen’s kappa. Secondary outcomes included the frequency and type of alternate surgical targets identified by DISE. The agreement on tonsil size between in-office physical assessment and DISE was also calculated. The effectiveness of DISE-directed surgery on postoperative S/SDB was not investigated.

**Results:**

Five hundred fifty-eight patients were included. DISE changed the surgical plan in 35% of patients. Agreement between DISE-based and AAP clinical practice guidelines-based management was low (κ = 0.354 +/- 0.021 [95% CI 0.312–0.395]). An alternate diagnosis or surgical target was identified by DISE in 54% of patients. There was moderate agreement on tonsil size (κ = 0.44 [0.33–0.55]) between DISE and in-office clinical assessment.

**Conclusions:**

This is a first phase diagnostic study, which demonstrates that DISE affects decision-making in surgically naïve children with S/SDB in up to 35% of patients. It has utility in individualizing first stage surgical treatments as well as identifying alternate targets for further surgical or medical therapy, while potentially limiting unsuccessful surgeries. Further studies to examine the effect of DISE on surgical outcomes are required.

## Background

Snoring/sleep disordered breathing (S/SDB) is a very common disease spectrum in children ranging from simple snoring to obstructive sleep apnea (OSA) with an overall prevalence of 4–11% [1, 2]. Depending on the severity, S/SDB can have detrimental health effects including behavioral issues, learning difficulties, educational underperformance, pulmonary hypertension, cor pulmonale, increased healthcare utilization and overall poor quality of life [1, 3–7]. In otherwise healthy children, adenotonsillar hypertrophy is widely accepted as the most common cause of S/SDB [3, 8–10]. Moreover, the American Academy of Pediatrics (AAP) endorses adenotonsillectomy (AT) as the first line treatment in all children with S/SDB in its clinical practice guidelines [4]. As a result, AT has become one of the most commonly performed surgical procedures in children in North America [3, 11, 12].

Using this treatment paradigm however, between 20 and 40% of patients will have persistent signs and symptoms of S/SDB after AT [2, 3, 10, 13]. Many of these patients will require further treatment, and in others AT (or one of its components) may have been unnecessary. Drug-induced sleep endoscopy (DISE) has been proposed to minimize treatment failures, tailor individual surgical treatments, and avoid unnecessary procedures. First described by Croft and Pringle in 1991 [14], DISE aims to dynamically evaluate the upper airway during simulated sleep. It is purported to identify obstruction and collapse at specific anatomic sites, thereby explaining the upper airway dysfunction leading to S/SDB [14].

DISE has been studied considerably in adults, with a smaller but growing body of literature in children. In adults, DISE findings and conditions have been claimed to accurately represent true sleep [15, 16], and to significantly affect management decisions in sleep surgery [17–19]. In children, the majority of work has been on patients who failed AT and/or special populations such as those with syndromes or neuromuscular abnormalities [20–25].

We believe that DISE is a useful tool in surgically naïve children serving as a roadmap for surgical planning to help eliminate needless operations, identify alternative surgical targets to AT, and in counseling parents on other options and the need for a secondary procedure or non-surgical treatment. To date, very little literature has addressed the use of DISE in this population. Only two studies claimed that the surgical decision was changed by DISE in up to 20% of patients [26], and that surgery was avoided in 11% of cases [27]. However, both studies were underpowered (25 and 37 patients respectively). Thus, the impact of DISE on surgical decision-making in that context is yet to be determined.

The purpose of this study was to determine the impact of DISE on surgical decision-making in surgically naïve children with S/SDB. This is the first important step in determining whether DISE delivers different or useful diagnostic information in comparison with the current paradigm [28]. We also aimed to evaluate the utility of DISE for identifying potential alternate surgical targets and avoiding surgical procedures in cases where they would not be beneficial.

## Methods

We conducted a retrospective observational cohort study at a tertiary pediatric Otolaryngology-Head & Neck Surgery referral center (Stollery Children’s Hospital, Edmonton, Alberta, Canada). Ethics approval was obtained from the University of Alberta Health Research Ethics Board (Pro00059158) prior to study commencement. The retrospective cohort was based on the records of a surgical database entered between January 2009 and December 2015.

Eligible patients were children age 3–17 diagnosed with S/SDB based on a score >33% on modified Pediatric Sleep Questionnaire (PSQ) [29], and an overnight pulse oximetry (PO) test. All patients underwent DISE-directed surgery for S/SDB. We excluded patients who underwent previous surgical management for S/SDB or other prior airway procedures, had the intended AT for one or more concomitant different diagnoses, craniofacial dysmorphism, syndromes or a neuro-muscular disorder.

Preoperative variables collected included demographics (age, sex), historical variables collected in the modified PSQ including duration of snoring, appropriate night time hygiene, short sleep onset, interruptions of sleep, sleep walking or talking, night terrors or nightmares, restlessness, night sweating, difficulty waking up in the morning, day time sleepiness, poor performance in education and parental smoking (inside or outside the house). Relevant past medical history was collected including obesity (growth percentile >97%), prematurity (<36 weeks gestation), neuropsychiatric diagnosis (general developmental delay, autism, attention deficit and hyperactivity disorder, etc.), prior intubation, allergy, asthma or wheezing, swallowing dysfunction or feeding difficulties, gastro-esophageal reflux disease (GERD), and family history of S/SDB. Physical examination findings were also collected including: dysmorphic features, tonsil size according to Brodsky scale [30], and adenoid size (1,<25%; 2,= > 25 or < 50; 3,= > 50% or <75%, and 4,= > 75% compromise of the nasopharynx) if nasopharyngoscopy was performed in the awake child [31], which is not done routinely on all patients with S/SDB. Approximately, 1 in 3 are scoped in clinic and they are usually at a certain older age group. Preoperative PO variables documented included mean oxygen saturation, oxygen saturation nadir, desaturation index and the McGill Oximetry Score (MOS) [32].

DISE was performed on all patients under a uniform sedation protocol using total intravenous anesthesia (TIVA). Currently, there is no uniform consensus on the optimal sedation protocol for DISE in children [33]. A consistent combination of remifentanyl (2–2.5 mcg/ml) and propofol (200–350 mcg/kg/min) was used for maintenance. If an inhalational anesthetic was used for induction, the tidal volume was ensured to be zero prior to performing DISE [8]. Lignocaine (1%) was used to topically anesthetize the nasal mucosa. DISE was performed following induction of anesthesia while the patient was spontaneously breathing. To ensure the appropriate plane for DISE, TIVA was titrated based on clinical response to stimulation, tolerance, reaction to endoscope insertion, vocal cord movement and regularity of abduction during inspiration. There is no standardized method to monitor TIVA in our institution, however. We aimed to achieve a level of sedation with spontaneous respirations (preferably with snoring), and enough endoscope tolerance to proceed with endoscopy. All endoscopies were digitally recorded and were sequentially maintained and available for review. A flexible neonatal bronchoscope (2.2 mm) was used to assess the airway (from the nasal cavity to the larynx). The scoring system used is summarized in Table 1. This system has been described in previous studies and has been shown to have good intra- and inter-rater reliability [8, 20, 34]. Nasal septal deviation (NSD) was graded on a 3 point scale (1: absent, 2:<50% compromise of nasal patency, 3:≥50% compromise of nasal patency), and the degree of rhinitis was graded according to a 3 point endoscopic rhinitis score (ERS) (Grade1, no obstruction to either side (mild or no rhinitis); grade 2, obstruction to 1 side; and grade 3, bilateral obstruction) [34].

**Table 1:** Scoring system for Drug-Induced Sleep Endoscopy (DISE)

Anatomic site/feature	Description	Score
ERS	No obstruction to either side, obstruction to one side, bilateral obstruction	1–3
NSD	None, <50%, >50%	1–3
Nasal polyps	No, Yes	0 or 1
Adenoids (retropalatal vs. choanal)	<25%, 25–50%, 50–75%, >75%	1–4
Tonsils	<50%, >50%	0 or 1
Pharynx	No collapse, lateral wall, circumferential, anteroposterior, tongue base	0 or 1
Lingual tonsil hypertrophy	No, Yes	0 or 1
Larynx	Normal, malacia, edema, paralysis/mobility disorder	0 or 1
Subglottic stenosis	No, Yes	0 or 1
Trachea	Malacia, stenosis, fistula	0 or 1
Bronchi	Malacia, stenosis	0 or 1

*ERS* Endoscopic rhinitis score, *NSD* Nasal septal deviation

### Outcome measures and statistical analyses

The primary outcome was to determine the agreement between the surgical decision based on DISE for all-comers who are surgically naïve and otherwise healthy, and the surgical decision based on the current AAP clinical practice guidelines, which recommends AT for all pediatric patients with S/SDB as first-line treatment. The secondary objectives were to report on the proportion of children with any type of alternate diagnoses identified by DISE in a surgically naïve patient population, which can be targeted in a second-stage procedure, as well as to determine the agreement on tonsil size between in-office physical assessment and DISE.

Basic descriptive statistics, standard deviations (SD), and 95% confidence intervals (CI) for each variable were calculated. Un-weighted Cohen’s kappa was used to determine the level of agreement between surgical decision based on DISE and the AAP recommendations. Agreement was defined as the same surgical decision based on DISE and AAP guidelines, whereas disagreement was defined as a different surgical decision based on DISE and the AAP guidelines. Significance was held at *p* <0.05. Statistics were performed on SPSS version 23.

## Results

In total, 1591 patients with S/SDB were identified retrospectively over a span of 7 years (January 2009 to December 2015). Of these, 932 patients underwent DISE-directed surgery. 423 were excluded (163 had previous surgical management of S/SDB, 98 were syndromic, 83 were out of the age range between 3–17, and 79 had a concurrent diagnosis of recurrent acute tonsillitis). 558 patients met all inclusion criteria and were included in the analysis.

Demographics of the patient cohort are included in Table 2. The mean age was 6.2 ± 2.7 years, with a slight male preponderance (59%). Obesity was present in 13%, and 10% had asthma. The rate of allergies was 12%, history of prematurity was 5%, and 4% had a neuropsychiatric diagnosis. The majority of patients (*n* = 396, 71%) had a MOS of 1 on preoperative PO (median MOS = 1). Parameters of the PO are included in Table 3.

**Table 2:** Demographics and Comorbidities

Parameters	Number of patients (%)
Mean Age (yrs) ± SD (range)	6.2 ± 2.7 (3.0–16.8)
Males	327 (59%)
Obesity	73 (13%)
Asthma	58 (10%)
Allergies	67 (12%)
Prematurity	28 (5%)
Neuro-psychiatric disorder	22 (4%)

**Table 3:** PO parameters

Parameter		Number of patients (%)
MOS	1	396 (71%)
	2	75 (13%)
	3	60 (10%)
	4	27 (5%)
Mean Desaturation Index ± SD (range)		5.92 ± 6.94 (0–73)
Mean SaO2 Saturation ± SD (range)		96.7 ± 1.25 (83–100)
Mean SaO2 Nadir ± SD (range)		86.8 ± 7.1 (51–98)

*MOS* McGill Oximetry Score

In total, DISE changed the surgical decision in 196 (35%) patients. The overall agreement between the AAP guidelines and the surgical decision based on DISE was only fair (κ = 0.354 +/- 0.021 [95% CI 0.312-0.395]). Of the 196 patients who did not undergo AT, 137 underwent adenoidectomy without tonsillectomy, 50 underwent tonsillectomy without adenoidectomy and nine had neither procedure. 45 patients underwent inferior turbinoplasty, and 5 patients underwent lingual tonsillectomy (Table 4).

**Table 4:** Procedures Performed in Patient Cohort

Procedure	Number of patients (%)
AT	362 (65%)
A without T	137 (25%)
T without A	50 (9%)
Endoscopic inferior turbinoplasty	45 (8%)
Lingual tonsillectomy	5 (1%)

*AT* adenotonsillectomy, *A* adenoidectomy, *T* tonsillectomy

An alternate diagnosis was identified by DISE in 303 (54%) patients. The most common alternate finding identified was pharyngeal collapse (lateral wall, circumferential, or anteroposterior tongue base), which was seen in 181 of the total patients (32%) and in 55 patients of the obese population (76%). In 78 patients, pharyngeal collapse was the only alternate diagnosis, the significance of which is uncertain. Other diagnoses included lingual tonsil hypertrophy (*n* = 39, 7%), laryngomalacia (LM) (*n* = 29, 5%), and obstructing NSD (*n* = 21, 4%). 123 patients (22%) had severe chronic rhinitis on ERS (Table 5). Together, these alternate diagnoses can provide an alternate medical or surgical target for a second-stage procedure or medical management. Noteworthy, a considerable number of patients were found to have adenotonsillar hypertrophy for which AT was undergone; and concurrently, an additional diagnosis such as pharyngeal collapse was established. For this reason, a discrepancy was demonstrated between the percentage of patients who had a surgical plan changed and those identified to have an alternate diagnosis based on DISE. There was moderate agreement on tonsil size (κ = 0.44 [0.33–0.55]) between DISE and in-office clinical assessment.

**Table 5:** Alternate Diagnoses Identified on DISE

Diagnosis	Number of Patients (%)
Pharyngeal collapse	181 (32%)
Severe rhinitis/inferior turbinate hypertrophy	123 (22%)
Lingual tonsil hypertrophy	39 (7%)
Laryngomalacia	29 (5%)
Severe nasal septal deviation	21 (4%)

## Discussion

This study reports on data of a homogenous group of children with S/SDB with no craniofacial malformation, syndromes or previous upper airway surgeries. Our results describe the basis of a more individualized surgical plan than the current standard. The results demonstrate that DISE-based decision-making changes the management from the traditional paradigm in more than one in three patients. Our results also demonstrate that in every other patient at least one alternate finding is found which may lead to persistent symptoms. This is significant given the fact that a large number of patients do not respond to AT, which may be due to collapse instead of actual obstruction.

Two previous studies have used DISE as a tool to tailor surgical management in the pediatric literature [26, 27]. Boudewyns et al. [27] were the first to report on DISE findings and treatment outcomes in surgically naïve children with S/SDB similar to our study population, i.e. without syndromes or craniofacial abnormalities. They performed a prospective study on 37 patients; their aims were to describe the pattern of upper airway obstruction found on DISE and evaluate the outcomes of DISE directed surgeries. All their patients underwent a pre and postoperative polysomnography (PSG). An apnea hypoapnea index (AHI) of less than 5 postoperatively was an indication of successful treatment. Based on DISE, 33 patients (89%) showed evidence of adenotonsillar obstruction. Of these, 28 underwent AT, while the remaining underwent either tonsillectomy or adenoidectomy alone. The four patients in that study who did not have evidence of adenotonsillar hypertrophy on DISE underwent medical management only. Overall, DISE changed the management from the traditional AAP-based paradigm in 9/37 (24%) patients, which is not significantly different from ours (35%), only that their sample size was considerably smaller. Another German study on 25 children similarly claimed that a 20% change in the initial management plan was observed [26]. Boudewyns et al. [27] also reported that findings other than adenotonsillar obstruction were found in 57% of patients, which is again in agreement with our study. Additionally, they also identified two patients (5%) with LM, consistent with our findings, but on the other hand, they did not find any lingual tonsil hypertrophy nor did they comment on significant chronic rhinitis. Their study reported a surgical success rate of 91% in the 22 patients who had PSG data available, indicating good outcomes based on DISE-directed surgery.

The remaining pediatric studies which used DISE to determine sites of upper airway obstruction in surgically naïve children were mainly in special populations such as syndromic patients [20, 35, 36]. Other studies included a mixture of surgically naïve children with S/SDB and those who were previously operated upon; yet they did not exclude syndromic patients as well [21, 22]. Galluzi et al. [37], conducted a systematic review of five papers that studied surgically naïve children undergoing AT and DISE (*n* = 39). They aimed to estimate the proportion of patients who had hypertrophy of tonsils and or adenoids. Upon eliciting a 62% rate (95% CI 44–79%), they did not feel that DISE had a utility in this group of patients. However, the significantly contaminated sample (Down syndrome, chronic lung disease, Pierre Robin, amongst others), with no analysis of a standard protocol for DISE nor alternate findings, calls for extreme caution on accepting the conclusion.

Identification of alternate diagnoses using DISE was variable in other studies. Truong et al. [22], studied children who underwent DISE directed surgery retrospectively, including both surgically naïve and those with persistent OSA after AT. In the latter group, lingual tonsillectomy was the most commonly preformed procedure due to obstruction at the tongue base, while AT and inferior turbinate cautery were the most commonly done in the surgically naïve group. Of note, 28% of patients in this group were hypotonic or syndromic children. Also, Wootten et al. [25] performed DISE-directed surgeries in children with refractory OSA. Similarly, the most frequently performed procedure was lingual tonsillectomy, and more than half of their patients (15/26) were syndromic. Their outcomes were determined based on variations in day and night symptoms, AHI, oxygen saturation nadir and post-operative improvement in airflow. Our data shows that lingual tonsillectomy was the least common procedure performed despite lingual tonsil hypertrophy being the third most common alternate diagnosis identified. Our explanation is that the surgical management would have potentially been performed at a later date as a second stage procedure following formally counseling parents about the identified surgical target.

A recent systematic review has shown 33–76% prevalence of persistent OSA following AT in obese children in comparison to 15–37% in the non-obese ones [38]. Interestingly, 76% of the obese patients in our study were found to have pharyngeal collapse. This may explain the cause behind high failure rates of AT in this specific patient population and how obesity maybe a predictor of surgical treatment failure.

Adenotonsillar hypertrophy as a cause of S/SDB has been thought of as a straightforward diagnosis. The Brodsky scale used in tonsil size assessment has moderate inter and intra-observer reliability [39], and taken with our findings that suggest only a moderate agreement between tonsil size based on Brodsky scale and DISE, it questions the decision of performing AT based on physical exam findings without definite dynamic assessment of the airway during sleep. The disagreement identified was not directional. In many patients, the DISE-based assessment was larger, and in some it was smaller. This is likely because in some patients, even large tonsils may not be obstructive during sleep, if they have good pharyngeal tone, are able to maintain oropharyngeal patency and the tonsil tissue does not extend into the hypopharynx. Whereas in others, even small tonsils may become obstructive when the pharynx is relaxed during sleep, and the inferior pole that is commonly hidden from view in clinic, extends into and obstructs the airway.

The limitations of our work include the retrospective study design, as well as the fact that this represented a single center experience, with a single surgeon performing and interpreting all endoscopies and DISE-directed surgeries, in a non-blinded fashion. In order to address these, a prospective study is currently underway, with DISE videos being interpreted by two separate Pediatric Otolaryngologists. The scoring system we used may not be used by other authors, which can limit comparisons and generalizability. However, it has been interpreted only in the pragmatic context of deciding on surgical treatment and there is no reasoned consensus around a single system thus far. We also believe that our consistent protocol utilizing the combination of propofol and remifentanyl is an advantage. The combination produces sleep like airway conditions at a reproducible assessment point, and reduces airway tone mediated by a reduction in genioglossus stimulation. This is also supplemented by clinical findings similar to PSG results found under normal sleep upon using propofol sedation [40]. Relatively recently, dexmedetomidine has been claimed to simulate natural muscle tone during sleep. In children with severe OSA based on PSG findings, a study demonstrated that an artificial airway was required in up to 57% of the patients sedated by propofol in comparison to 7% in the dexmedetomidine group, which may imply that dexmedetomidine did not resemble PSG findings as closely as propfol [41]. However, some authors use this evidence to say that propofol use results in exaggerated relaxation beyond what is found in natural sleep. In the adult OSA population, patients undergoing propofol sedation for DISE had a significantly increased likelihood of demonstrating complete tongue base obstruction (75%) as compared with the dexmedetomidine group (42.7%), which significantly affected the configuration of upper airway obstruction witnessed during DISE [42]. As of yet, there is no agreement on an ideal anesthetic regimen for DISE [33] and a combination of propofol and remifentanyl was used for all our patients to ensure uniformity.

Ideally, rapid eye movement (REM) sleep is the stage we aimed to mimic, due to most obstructive events occurring during this stage. Nonetheless, DISE was not performed with a PSG simultaneously. Therefore, the exact stage of simulated sleep achieved is unknown. Determining the depth of anesthesia and ensuring that the anesthetic-induced sleep is an appropriate representation of true sleep has been a challenge for DISE, and has been examined in other studies [15, 16, 43, 44]. In adults, PSG data as well as Bispectral Index (BIS) monitoring has shown that DISE using a combination of propofol, midazolam and/or narcotics can achieve a state of sedation nearly identical to natural sleep [15, 16].

Our work also suffered from the disadvantage of the absence of PSG data. However, we used the most pragmatic criteria at the disposal of the otolaryngologist in mainstream practice (the PSQ and PO). It is of relevance to mention that in certain practices, neither overnight PO nor PSG is performed on all patients with S/SDB. As a matter of fact, the decision to perform AT relies solely on clinical history and non-specific physical exam findings. While PSG is known to be the reference standard for diagnosis, the current American Academy of Otolaryngology Head and Neck Surgery guidelines for tonsillectomy state that the use of PSG is not always necessary and that history and physical examination should be the initial approach [45]. In our study population, 71% of the patients had a MOS of 1, which is inconclusive or normal. However, the overnight PO is only a screening test with a low negative predictive value. As a result, a MOS of 1 does not rule out S/SDB and parents should not be reassured. In fact, children may even suffer from significant disease [32]. In addition, all patients included in our study scored positive on the modified PSQ, which is also used when a PSG is not practical. At our center, PO is primarily used to determine the safest post-operative environment for the patient (i.e. day surgery, overnight stay with routine care, or intensive care unit monitoring) and has been shown to have excellent utility when being used in this fashion [32]. This study did not focus on outcomes and success rates. Nevertheless, unpublished data from our center shows that PO normalization following AT does not signify symptom resolution and PSQ is the determining factor.

Further, we have represented our results in terms of agreement statistics, which put in perspective the gross degree of discordance between the decisions rather than simply an expression in percentage that is subject to chance. Finally, we only compared DISE-based surgical decision-making with the AAP recommendations, which we concede that not all clinicians adhere to literally. Some surgeons would argue that if the tonsils were very small, they would not consider tonsillectomy for S/SDB. However, due to the variability in practice surrounding this point, we chose to use our reference standard as the AAP guidelines because these are in fact the only published set of guidelines on when to remove tonsils for S/SDB, and they do not consider size. In fact, trans-oral tonsil size has been shown to have a poor correlation with severity of S/SDB [46].

We believe that studying surgically naïve children by DISE is a well overdue step in this research field. Limiting DISE substrate to only complex patients deprives the experience from the full spectrum of findings and pathology and excludes the less affected and normal patients in contrast to standards of evidence-based diagnostic research [47]. In the future, we plan to perform a prospective observational study of DISE-directed surgery in surgically naïve children to further validate the findings presented here. We also aim to examine symptom-based and objective outcomes in patients undergoing DISE-directed surgery vs. traditional surgery for S/SDB and their impact on post surgery DISE, ideally in a randomized controlled trial as a phase 3 diagnostic research study. This would allow us to see the full effect of decision change, and whether or not DISE- directed surgery is superior, similar or inferior. Additionally, we hope to perform a formal cost analysis to determine the cost effectiveness of DISE including the number and type of procedures that can potentially be avoided.

## Conclusions

DISE affects decision-making in surgically naïve children with S/SDB in up to 35% of patients. It has utility in individualizing first stage surgical treatments as well as identifying alternate targets for further surgical or medical therapy, while potentially limiting unsuccessful surgery. Further research is needed to determine if and how DISE affects clinical outcomes in pediatric sleep surgery.

## Abbreviations

AAP: American academy of pediatrics
AHI: Apnea hypoapnea index
AT: Adenotonsillectomy
BIS: Bispectral index
CI: Confidence interval
DISE: Drug-induced sleep endoscopy
ERS: Endoscopic rhinitis score
GERD: Gastro-esophageal reflux disease
LM: Laryngomalacia
MOS: McGill oximetry score
NSD: Nasal septal deviation
OSA: Obstructive sleep apnea
PO: Pulse oximetry
PSG: Polysomnography
PSQ: Pediatric sleep questionnaire
REM: Rapid eye movement
S/SDB: Snoring/sleep disordered breathing
SD: Standard deviation
TIVA: Total intravenous anesthesia
